# The Nuclear Pore Complex Mediates Binding of the Mig1 Repressor to Target Promoters

**DOI:** 10.1371/journal.pone.0027117

**Published:** 2011-11-14

**Authors:** Nayan J. Sarma, Thomas D. Buford, Terry Haley, Kellie Barbara-Haley, George M. Santangelo, Kristine A. Willis

**Affiliations:** Department of Biological Sciences, The University of Southern Mississippi, Hattiesburg, Mississippi, United States of America; Brunel University, United Kingdom

## Abstract

All eukaryotic cells alter their transcriptional program in response to the sugar glucose. In *Saccharomyces cerevisiae*, the best-studied downstream effector of this response is the glucose-regulated repressor Mig1. We show here that nuclear pore complexes also contribute to glucose-regulated gene expression. NPCs participate in glucose-responsive repression by physically interacting with Mig1 and mediating its function independently of nucleocytoplasmic transport. Surprisingly, despite its abundant presence in the nucleus of glucose-grown *nup120*Δ or *nup133*Δ cells, Mig1 has lost its ability to interact with target promoters. The glucose repression defect in the absence of these nuclear pore components therefore appears to result from the failure of Mig1 to access its consensus recognition sites in genomic DNA. We propose that the NPC contributes to both repression and activation at the level of transcription.

## Introduction

Glucose is the preferred carbon source of almost all life on earth. Defective glucose metabolism is linked to a number of human diseases, the most prominent of which are metabolic syndrome and diabetes. A central player in the maintenance of glucose homeostasis is the AMP-activated protein kinase, or AMPK. AMPK modulates the secretion of insulin by pancreatic β-cells, and is the target of metformin, a drug frequently used in the treatment of diabetes [1], [2], [3]. AMPK carries out its function by phosphorylating multiple cytoplasmic enzymes, but it also participates directly in the regulation of gene expression by phosphorylating multiple different transcription factors [4], [5], [6], [7].

The model eukaryote *Saccharomyces cerevisiae* is an ideal choice for the study of glucose metabolism and glucose-regulated gene expression for two main reasons. First, AMPK, its activating kinase LKB1, and many of the proteins that mediate the response to glucose are highly conserved between *S. cerevisiae* and humans. Second, *S. cerevisiae* has a uniquely fermentative lifestyle, meaning that yeast cells are optimally evolved for the efficient metabolism of glucose. Our current understanding of the glucose response and glucose-regulated gene expression in *S. cerevisiae* has been established largely through studying the regulation of the *SUC2* gene, which codes for the easily assayable enzyme invertase.

Work completed over the past twenty-five years has identified numerous proteins that are required to control transcription of *SUC2*, although their means of action has remained at least partly unclear. Under conditions that repress *SUC2* expression, defined as growth in the presence of glucose, the AMP kinase homolog Snf1 is inactive, and transcription of *SUC2* is repressed by the DNA binding protein Mig1 [8], [9]. When glucose is withdrawn or depleted, the LKB1 homologs Sak1, Elm1, and Tos3 phosphorylate and activate Snf1, which then enters the nucleus and phosphorylates Mig1. The phosphorylated repressor is exported from the nucleus, allowing transcriptional initiation to occur. Another transcriptional regulator, Gcr1, binds to the *SUC2* promoter at a position immediately adjacent to Mig1. Deletion of *GCR1* causes a general defect in the regulation of *SUC2* transcription, as it both impairs repression of the gene in the presence of glucose and reduces its expression in the absence of glucose [8], [10]. The Swi/Snf chromatin remodeling complex, the SAGA histone acetyltransferase complex, and the RNA polymerase II elongation factor Spt6 [11], [12], [13], [14], [15], [16], [17], [18], [19], [20] are also required for transcription of *SUC2*.

Multiple subunits of the nuclear pore complex (NPC) have also been shown to interact constitutively with the *SUC2* promoter [21], and recent evidence has suggested that NPCs play a central role in transcriptional regulation of eukaryotic gene expression. Regulatory control of many human loci appears to involve contact with the Nup93 subunit of the NPC [22], [23], while artificial tethering of human genes to the inner nuclear membrane results in transcriptional activation of some genes and repression of others [24]. Interestingly, NPCs in *S. cerevisiae* have boundary activity, allowing them to separate regions of active and repressed chromatin [25].

We report here the involvement of specific subunits of the NPC in regulation of *SUC2* expression. The effect of these nucleoporins on repression appears to be mediated by Mig1, which physically associates with NPCs. In the absence of either of two nucleoporins, Nup120 or Nup133, nucleocytoplasmic transport of Mig1 is unaltered, but the ability of the repressor to co-purify with intact NPCs is severely impaired. Surprisingly, despite its abundant presence in the nuclear lumen of glucose-grown *nup120*Δ and *nup133*Δ cells, Mig1 has lost its ability to interact with target promoters. The glucose repression defect in the absence of these two subunits of the Nup84 subcomplex therefore appears to result from the failure of Mig1 to access its consensus recognition sites in genomic DNA.

## Results

### Identification of nucleoporins that contribute to regulation of SUC2

**Figure 1:**
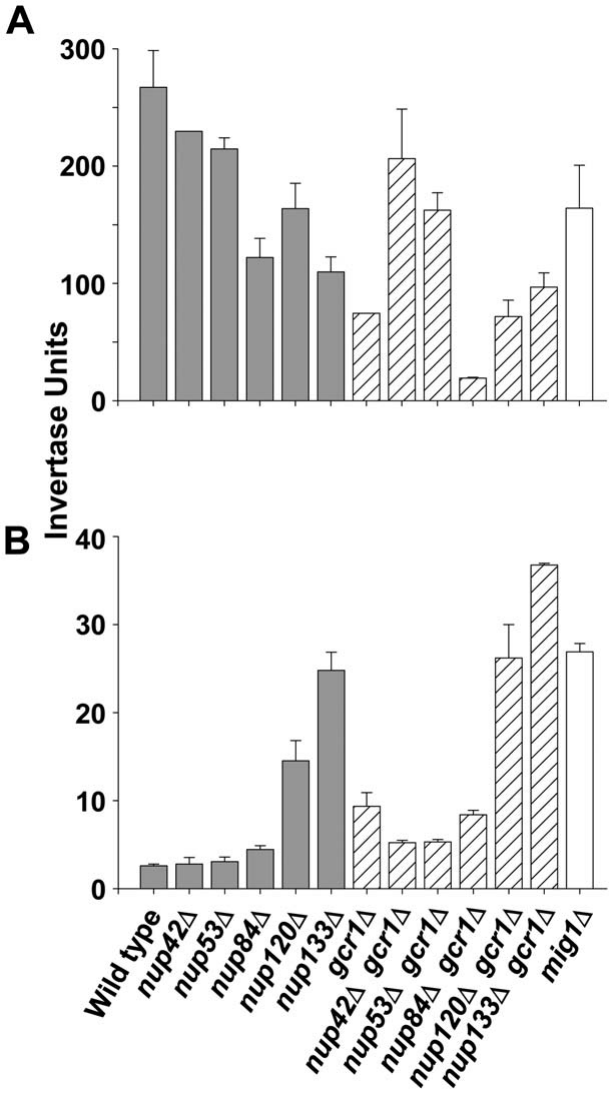
Different nucleoporins make specific contributions to regulation of *SUC2* expression. Invertase activity in wild type (WT) and mutant strains grown under either de-repressing (A) or repressing (B) conditions. Error bars represent the standard error of the mean for four independent determinations.

In our previous work, we showed that components of the NPC physically interact with the *SUC2* promoter when it is both repressed and de-repressed [21]. To determine whether this association reflects a role for nucleoporins in regulating the expression of this canonical glucose-regulated gene, we first assayed levels of invertase, the easily detected *SUC2* product [26], in a series of strains that each lacked an NPC subunit or NPC-associated factor. As expected, deletion of *NUP42*, which is localized exclusively to the cytoplasmic side of the NPC, has no substantial effect on regulation of *SUC2* expression (filled bars, Fig. 1A, B and Table 1). Deletion of *NUP53* also has no substantial effect on regulation of *SUC2* (filled bars, Fig. 1A, B and Table 1), despite the fact that the *NUP53* gene product ChIPs to the *SUC2* promoter in wild type cells [21]. Deletion of *NUP84* has only a minor effect on regulation (filled bars, Fig. 1A, B and Table 1); these cells exhibit an approximately 40% decrease in invertase production when grown under de-repressing conditions (filled bar, Fig. 1A), but their ability to repress *SUC2* transcription is almost normal (filled bar, Fig. 1B). Deletion of either *NUP120* or *NUP133* results in minor defects in invertase production under de-repressing conditions, comparable to that seen in the absence of *NUP84* (filled bars, Fig. 1A). However, unlike other nucleoporins, deletion of either *NUP120* or *NUP133* results in severe defects in repression of *SUC2* (Fig. 1B, Table 1). In the case of *NUP133* deletion, the defect in repression is as severe as elimination of Mig1 itself (open bar, Fig. 1B).

**Table 1:** Deletion of nucleoporins compromises regulation of *SUC2* expression.

Genotype	D∶R ratio^a^
Wild type	102.2
*nup42*Δ	82.1
*nup53*Δ	69.9
*nup84*Δ	27.4
*nup120*Δ	11.3
*nup133*Δ	4.4
*gcr1*Δ	8.0
*gcr1*Δ *nup42*Δ	39.5
*gcr1*Δ *nup53*Δ	30.6
*gcr1*Δ *nup84*Δ	2.3
*gcr1*Δ *nup120*Δ	2.7
*gcr1*Δ *nup133*Δ	2.6

a Ratio of invertase activity in derepressed and repressed conditions, a measure of the regulation of *SUC2* expression, calculated from absolute units of invertase activity presented in Figure 1.

Like Nup120 and Nup133, the DNA-binding transcription factor Gcr1 affects both repression and derepression of *SUC2* (hatched bars, Fig. 1A, B, Table 1 and [10], [27], [28], [29]). Since Gcr1 also physically associates with NPCs [30], we thought these nucleoporins might affect regulation of *SUC2* by working through Gcr1. To test this idea, we introduced the *gcr1*Δ lesion into cells already carrying deletions of *NUP42*, *NUP53*, *NUP84*, *NUP120*, or *NUP133* and assayed for invertase. Surprisingly, deletion of either *NUP42* or *NUP53* appears to partially suppress the defect in *SUC2* regulation caused by deletion of *GCR1*. Cells deleted for both *NUP84* and *GCR1* display a synthetic defect in invertase production (hatched bars, Fig. 1A), but no synthetic defect in repression (hatched bars, Fig. 1B). Conversely, *nup120*Δ *gcr1*Δ and *nup133*Δ *gcr1*Δ double mutants display no substantial synthetic defect in invertase production (hatched bars, Fig. 1A), but have a clear synthetic defect in *SUC2* repression (hatched bars, Fig. 1B) that is at least equivalent to removal of Mig1 itself (Fig. 1B, open bar). These synthetic defects suggest that rather than working together, Gcr1 and NPCs likely operate in parallel pathways that make distinct contributions to the regulation of *SUC2*.

### Nucleocytoplasmic transport of Mig1 is normal in the absence of Nup120 or Nup133

NPCs are now known to participate in multiple steps of gene regulation, including initiation, splicing, termination, and mRNA export [31]. Since an increase in levels of invertase is not easily explained based on defective splicing, termination, or export of *SUC2* mRNA, we chose to focus on understanding the cause of the defect in *SUC2* repression that we observed in the absence of either *NUP120* or *NUP133*. We previously used Quantitative Fluorescent Protein Detection (QFPD), a novel assay for the sensitive and quantitative measurement of fluorescently tagged protein levels, to demonstrate that Mig1 exhibits a regulated association with NPCs, co-purifying only under conditions where it functions to repress transcription [21]. Furthermore, deletion of *HXK2* both impairs repression and eliminates NPC association without disrupting nuclear localization of the repressor [21]. We therefore considered the possibility that Nup120 and Nup133 contribute to repression of *SUC2* through Mig1.

**Figure 2:**
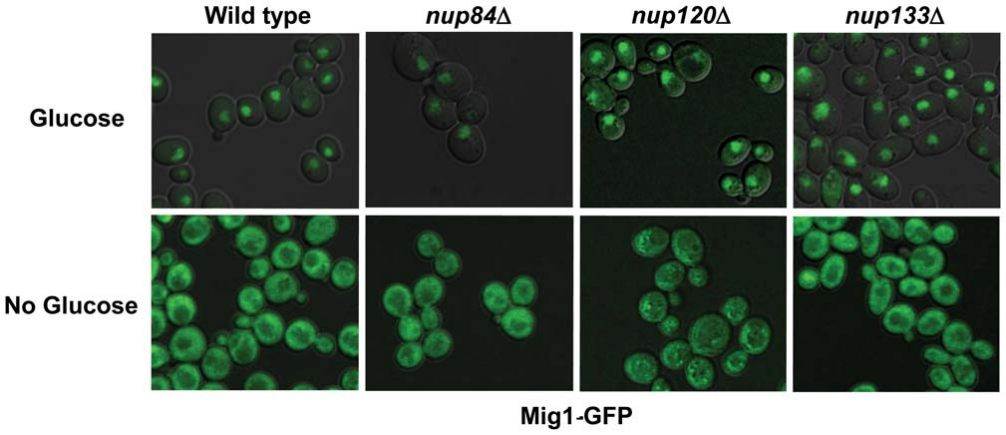
Nucleocytoplasmic shuttling of Mig1 occurs normally in the absence of *NUP120* or *NUP133*. Confocal images show localization of Mig1-GFP in either the presence (top panels) or absence (bottom panels) of glucose, in either wild type (WT) or mutant strains.

Mig1 is imported into the nucleus only in the presence of glucose [32], [33]; in other words, localization of the repressor correlates with its function. Since NPCs have a well-established role in nucleocytoplasmic transport [34], [35], [36], it was therefore crucial to first test the hypothesis that deletion of *NUP120* or *NUP133* interferes with *SUC2* repression by impairing nuclear localization of Mig1. To check this possibility, we used confocal fluorescence microscopy to observe the localization of our fully functional GFP-tagged allele of Mig1 in *nup84*Δ, *nup120*Δ, and *nup133*Δ cells. Consistent with previous reports that Nup120 and Nup133 do not affect nucleocytoplasmic transport of proteins [37], [38], localization of Mig1-GFP (Fig. 2) and Snf1-GFP (not shown) occurs normally in the absence of each of these three nucleoporins. We therefore conclude that the loss of repression we observe in *nup120*Δ and *nup133*Δ cells is not an indirect consequence of defective transport.

### In the absence of Nup120 or Nup133, perinuclear compartmentalization of Mig1 is lost

**Figure 3:**
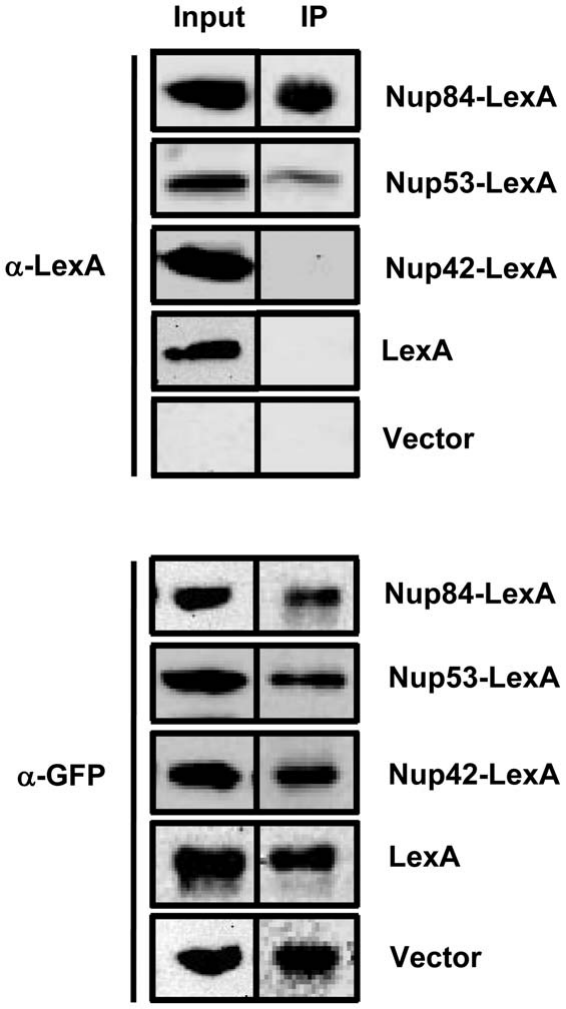
Mig1 interacts with the Nup84 subcomplex. First lane (Input) shows the presence of the expressed proteins in the cell lysates; second lane (IP) shows the presence or absence of LexA-tagged proteins in the immunoprecipitated samples (top panel), and the immunoprecipitation of GFP-tagged Mig1 with the anti-GFP antibody in all the conditions tested (bottom panel). Vector, sample without any LexA-tagged protein.

The above data are consistent with the idea that defective *SUC2* repression in the absence of Nup120 or Nup133 might stem from impaired targeting of Mig1 to the nuclear periphery, analogous to the defect we previously observed to occur in the absence of Hxk2 [21]. To test this idea, we first asked whether Mig1 is capable of interacting with the highly stable subcomplex within the nuclear pore that contains both Nup120 and Nup133. We chose to evaluate association with Nup84, since it is part of this same complex but its deletion does not appear to seriously compromise Mig1 function, as judged by the near normal repression of invertase in glucose-grown *nup84*Δ cells (Fig. 1B). We therefore immunoprecipitated a fully functional Mig1-GFP fusion protein from glucose grown cells in which Nup84 was tagged with a lexA epitope. This lexA fusion complements deletion of *NUP84*, and the further addition of YFP to the C-terminus of the chimera shows that it correctly localizes to the nuclear periphery (Fig. S1). α-GFP antibody was added to crude lysate, then protein A sepharose was added to pull down those antibody/Mig1-GFP complexes. Antibody-sepharose slurries were washed as previously described [39], then the adhering proteins were eluted, blotted, and probed with α-lexA antibody. As a control, blots were also probed with α-GFP to ensure that an equal amount of Mig1 was pulled down in all conditions. We found that Nup84-lexA does co-immunoprecipitate with Mig1-GFP. Relative to Nup84, there is a greater than two-fold reduction in co-immunoprecipitation of Nup53-lexA by Mig1-GFP, consistent with the negligible effect that deletion of *NUP53* has on *SUC2* regulation (Fig. 1 and Table 1). Neither the cytoplasmic nucleoporin Nup42 fused to lexA nor lexA alone co-immunoprecipitates with Mig1 (Fig. 3). Our data therefore confirm that Mig1 associates with NPCs under conditions where it represses transcription of its target genes (Fig. 3). Furthermore, this association appears specific to the nuclear side of the pore and is stronger with Nup84 than with Nup53, consistent with a role for the Nup84 subcomplex in regulation of *SUC2* expression.

**Figure 4:**
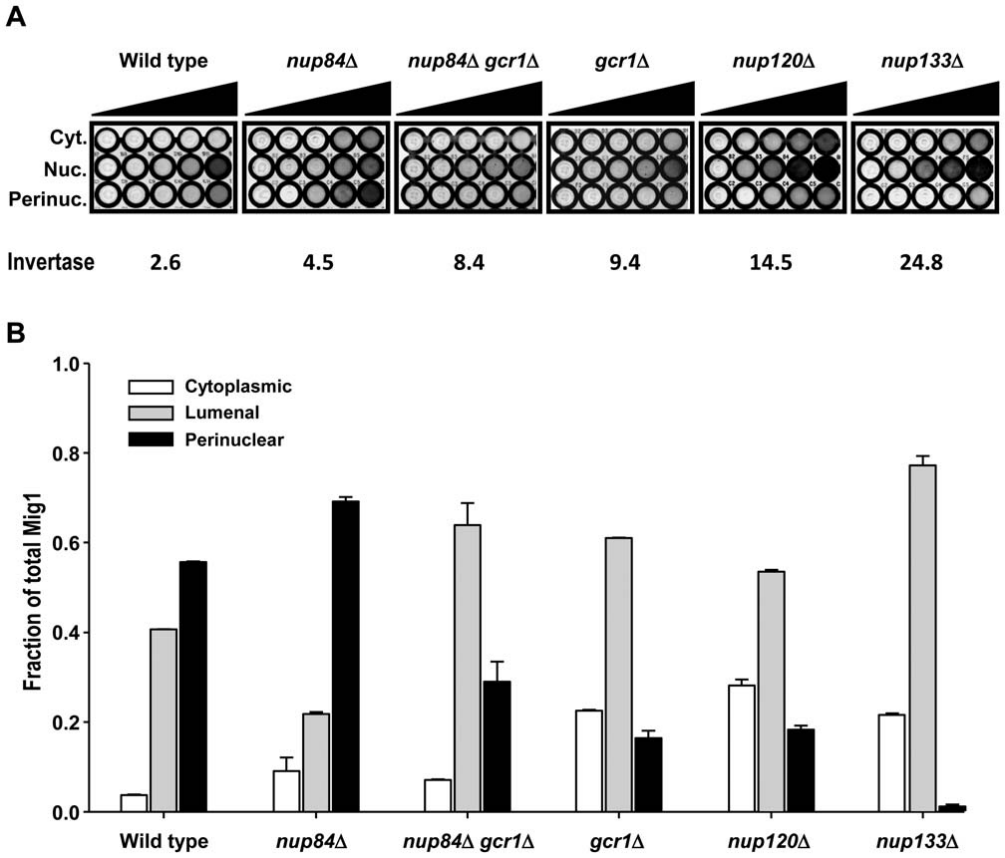
Levels of perinuclear, but not nuclear or total cellular, Mig1, correlate with repression of *SUC2*. (A) Quantitative fluorescent protein detection (QFPD) of Mig1-GFP in repressing conditions. Increasing amounts of protein from cytoplasmic, nuclear (perinuclear+lumenal), and perinuclear fractions isolated from wild type or mutant strains were loaded into microtiter wells (circles, left to right); fluorescence was measured as described in Materials & Methods. Units of invertase, also in repressing conditions, are shown for comparison. (B) Densitometric analysis of the data shown in A. The fraction of Mig1-GFP present in the cytoplasm (cytoplasmic; open bars), nuclear lumen (lumenal; shaded bars), and perinuclear compartment (perinuclear; filled bars) is shown for each strain. Error bars represent the standard error of the mean.

**Figure 5:**
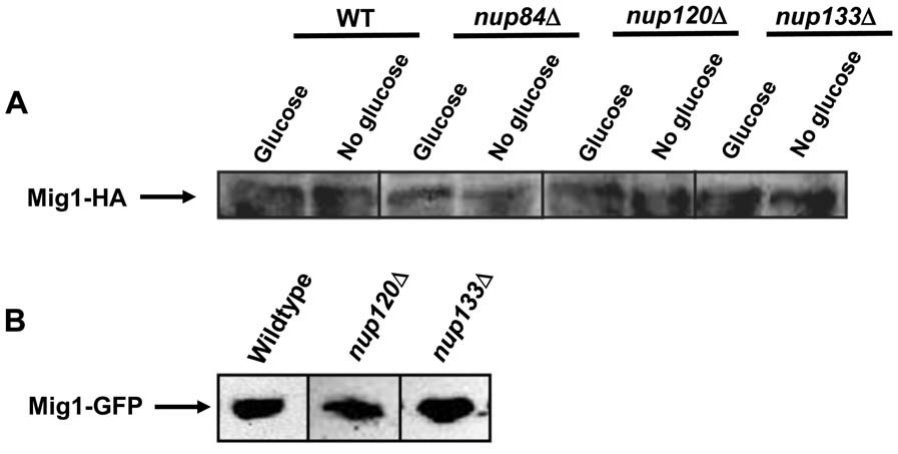
Levels of the Mig1 protein are not reduced in the absence of *NUP120* or *NUP133*. (A) Levels of HA-tagged Mig1 in crude lysate isolated from wild type, *nup84*Δ, *nup120*Δ, and *nup133*Δ cells grown in media containing glucose (repressing conditions) or pyruvate (derepressing conditions) as the carbon source. (B) Levels of GFP-tagged Mig1 in crude lysate isolated from wild type, *nup120*Δ, and *nup133*Δ cells grown in media containing glucose as the carbon source (repressing conditions). 100 µg total protein in each lane.

We next introduced the same *MIG1-GFP* allele into the *nup84*Δ, *nup120*Δ, *nup133*Δ, *gcr1*Δ, and *nup84*Δ*gcr1*Δ mutants, and used QFPD to measure co-fractionation of fluorescent Mig1 with perinuclear factors. In a *nup84*Δ mutant, which displayed near normal *SUC2* repression (Fig. 1B), there was no decrease in the percentage of Mig1 that co-fractionated with NPCs (Fig. 4 and Table 2). In all other mutants tested, there was a strong correlation (R^2^ = 0.93) between loss of *SUC2* repression, shown as an increase in invertase levels, and the fraction of Mig1 associated with NPCs (Fig. 4, Table 2); as Mig1 is lost from the perinuclear subcompartment, inhibition of *SUC2* expression is lost exponentially (Fig. S3). This was not due to an overall reduction in levels of the Mig1 protein, which were no lower than in wild type cells (Fig. 4, Fig. 5, and Table 2). In fact, there was an inverse relationship (R^2^ = 0.72) between the amount of Mig1 present in the nucleus of these mutants and the degree of *SUC2* repression (Table 2). Student's t-test indicates that the difference between the slopes of the curves that describe these relationships is highly significant (t = 3.55, p = 0.01). Indeed, though there appears to be four-fold more Mig1 in the nuclear lumen of *nup120*Δ and *nup133*Δ cells, its capacity to function as a repressor is severely impaired. These data suggest that defective glucose repression upon removal of perinuclear factors results from impaired subnuclear targeting of the Mig1 repressor.

**Table 2:** Repression of *SUC2* correlates with subnuclear targeting of Mig1.

		NUCLEAR Mig1^a^		
	*% SUC2*repression^b^	Perinuclear	Lumenal	Total^c^
WT	100	1.00	1.00	1.00
*nup84*Δ	58	1.30	0.57	1.47
*gcr1*Δ *nup84*Δ	31	0.53	1.63	1.26
*gcr1*Δ	28	0.36	1.87	1.53
*nup120*Δ	18	0.43	1.77	2.48
*nup133*Δ	10	0.03	2.33	2.22

a Amount of Mig1 in each nuclear fraction (perinuclear, lumenal, or total) relative to wild type (WT), which was in each case set to 1.00.

b Invertase levels under repressing conditions; each mutant is shown as a percentage of WT. Error is less than or equal to 10%.

c Total = Perinuclear+Lumenal.

### Lumenal Mig1 is unable to bind its target promoters

**Figure 6:**
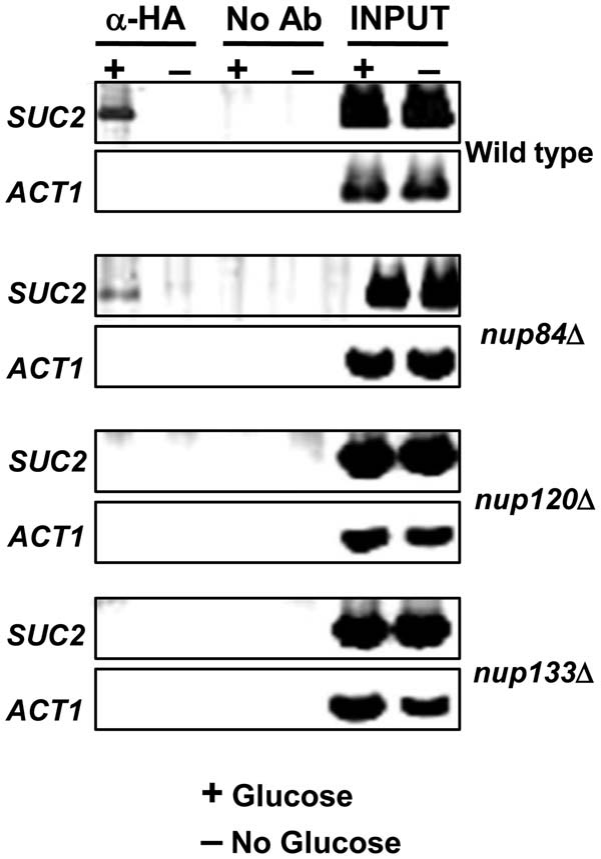
Mig1 fails to bind its target site in the *SUC2* promoter in the absence of *NUP120* or *NUP133*. HA-tagged Mig1 (α-HA) was immunoprecipitated from wild type (A), *nup84Δ* (B), *nup120Δ* (C), and *nup130*Δ (D) cells grown in either the presence (+) or absence (−) of glucose. PCR was used to amplify the promoters of *SUC2* and *ACT1* (negative control) from immunoprecipitated material (α-HA), no antibody negative control (No Ab), and whole cell extracts (Input).

Although QFPD shows that perinuclear targeting of Mig1 is impaired in the absence of either Nup120 or Nup133 (Fig. 4 and Table 2), this *in vitro* analysis agrees with our *in vivo* demonstration (Fig. 2B) that the repressor is localized to the nucleus. However, nuclear localization does not automatically denote DNA binding, so loss of Mig1 interaction with its target promoters was among the possible explanations for the global impairment of glucose repression that we observed. We tested this hypothesis by using ChIP to measure *in vivo* Mig1 binding to the *SUC2* promoter in both the presence and absence of glucose; linearity of the PCR reaction was confirmed over a three-fold range of template (Fig S3). The *ACT1* promoter was used as a control because it lacks a Mig1 binding site. As shown previously [21], in wild type cells Mig1 is bound to the *SUC2* promoter only in the presence of glucose (Fig. 6A). In glucose-grown *nup84*Δ cells, where subnuclear targeting of Mig1 to the perinuclear compartment is unimpaired (Fig. 4 and Table 2), crosslinking of Mig1 to the *SUC2* promoter was almost as efficient as in wild type cells (Fig. 6B). Surprisingly however, in glucose-grown *nup120*Δ or *nup133*Δ cells, where Mig1 is depleted from the perinuclear subcompartment but is abundantly present in the nuclear lumen (Fig. 2, Fig. 4, and Table 2), Mig1 binding to the *SUC2* promoter is undetectable (Fig. 6C, D). This suggests that interaction with NPCs is required for Mig1 to gain access to its consensus binding site in the *SUC2* promoter (Table 3).

**Table 3:** Binding of Mig1 to glucose-repressed promoters.

Gene	Position^a^	% binding in*nup120*Δ	% binding in*nup133*Δ
*SUC2*	−498	2.3	0.9
*HXK1*	−727	1.0	1.1
*HXT4*	−465	18.6	20.6
*TPS1*	−269	2.5	2.4

a Location of the consensus Mig1 site relative to the start codon of each gene.

**Figure 7:**
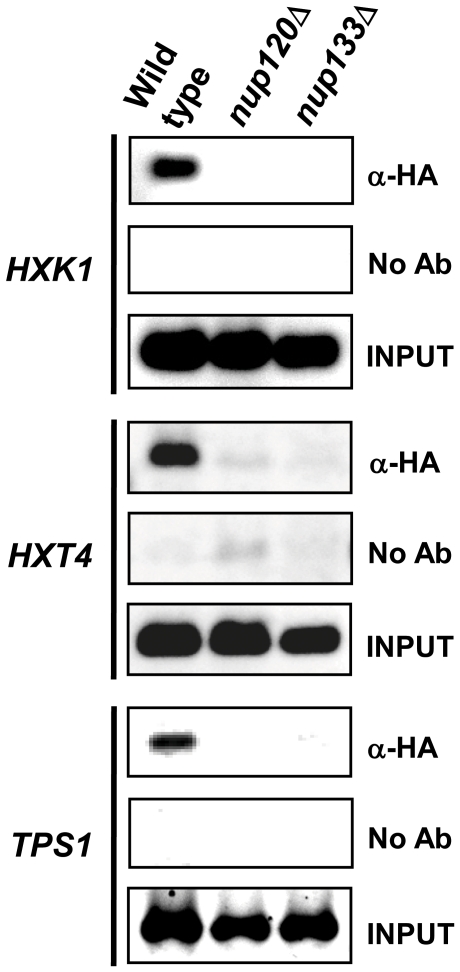
Deletion of *NUP120* or *NUP133* eliminates Mig1 binding to additional target promoters. HA-tagged Mig1 (α-HA) was immunoprecipitated from wild type, *nup120Δ*, and *nup130Δ* cells grown in the presence of glucose. PCR was used to amplify the promoters of *HXK1*, *HXT4*, and *TPS1* from immunoprecipitated material (α-HA), no antibody negative control (No Ab), and whole cell extracts (Input).

We did ChIP analysis of several other verified Mig1 target promoters to test whether the failure of nuclear Mig1 to recognize its consensus DNA binding site in the absence of Nup120 or Nup133 is unique to the *SUC2* promoter. We tested three other Mig1 target promoters, and found that in glucose-grown cells lacking either Nup84 subcomplex component, Mig1 binding is dramatically impaired (Fig. 7 and Table 3). Each of these genes (*HXK1*, *HXT4*, and *TPS1*) is known to contain a functional upstream consensus binding site for the Mig1 repressor [40] and to be transcriptionally repressed by Mig1 in glucose-grown cells [41]; our microarray analysis confirms that *HXK1*, *HXT4* and *TPS1* are up-regulated in *nup120*Δ or *nup133*Δ mutants grown under repressing conditions (data not shown).

## Discussion

We previously established that glucose repression of *SUC2* requires targeting of the Mig1 repressor to the nuclear pore complexes [21]. Since this implicated NPC subunits or components of the nuclear basket [42] in Mig1 function, we tested the glucose repression mechanism in multiple mutants, each deleted for a different perinuclear factor. We found that deletion of the transcription factor *GCR1*, of a specific subset of NPC subunits, or of both in combination resulted in substantial defects in the regulation of *SUC2* (Fig. 1A, B). Double deletions of *GCR1* and certain nucleoporin genes resulted in a synthetic regulatory defect; presumably such synthetic defects in gene regulation also contribute to the synthetic growth phenotype of *gcr1*Δ *nup*Δ double mutants [30]. Many of the mutants that we tested displayed defects in both repression and derepression of *SUC2*. This result is not surprising. Gcr1 and its extensively studied perinuclear interaction partner Rap1 are well known to function as both repressors and activators of transcription [10], [27], [28], [39], [43], [44], [45], [46], [47], while components of the yeast NPC can block the spread of heterochromatin, thus defining boundaries between active and repressive regions of the genome [25]. Ultimately, a solution to the long-standing puzzle of how a single protein can function as both repressor and activator may require further consideration of NPC-mediated nuclear organization as a significant factor in the regulation of gene expression [46].

Since NPCs participate in multiple steps of gene expression, derepression defects in *nup* mutants are somewhat difficult to interpret; the decreased invertase levels we observed may reflect a compound defect in transcription initiation, RNA processing, and/or mRNA export. However, the repression defects we see are simpler to understand. A problem in processing or exporting the *SUC2* mRNA would not result in higher levels of invertase, and we have shown here that import of Mig1, which blocks expression of *SUC2*, is not impaired in *nup* mutants with repression defects (Fig. 2). It therefore seems likely that the repression defects we see reflect a role for NPCs in the regulation of gene expression at the level of transcription.

We show here that specific subunits of the NPC co-immunoprecipitate with Mig1 in wild type cells. The cytoplasmic Nup42 is not co-immunoprecipitated by Mig1, suggesting that the physical association between the pore and the repressor occurs exclusively on the nuclear side of the envelope. This is not the result we would expect if interaction was solely for the purpose of transporting Mig1 from the cytoplasm through the pore and into the nucleus. Nup84 is co-immunoprecipitated by Mig1, and Nup53 co-immunoprecipitates weakly. This is generally consistent with our observation that deleting components of the Nup84 subcomplex has an effect on repression of *SUC2*, while deleting *NUP53* does not. However, it should be noted that no subunit found on the nucleoplasmic face of the pore is likely to yield a completely negative result in this assay, since the NPC as a whole is stable to biochemical purification.

We chose to focus our study on two strains, those carrying lesions in either *NUP120* or *NUP133*, where glucose repression was severely impaired. In these mutants, we found that both the total amount of Mig1 and the amount of Mig1 in the nucleus were equal to or greater than in wild type (Fig. 4, Fig. S2, and Table 2). Despite this, Mig1 co-purified with NPCs in wild-type (Fig. 4 and [21]) but not *nup120*Δ or *nup133*Δ cells (Fig. 4 and Table 2). In glucose-grown *nup133*Δ cells, where only 3% of Mig1 co-fractionated with NPCs, *SUC2* expression was increased ten-fold, i.e. removal of Nup133 or Mig1 results in an approximately equivalent defect in glucose repression. Conversely, in *nup84*Δ cells, which had at most a mild defect in *SUC2* regulation, Mig1 co-fractionation with NPCs was unimpaired. Further work is needed to explain the observation that Nup120 and Nup133 have a greater effect on the function and subnuclear targeting of Mig1 than do other nucleoporins; the difference from Nup84, which is part of the same NPC subcomplex as Nup120 and Nup133, is especially intriguing. One obvious possibility is the impact of the pronounced NPC clustering observed in *nup120*Δ and *nup133*Δ cells ([48] and our unpublished data). Unfortunately little is yet known about the underlying cause of NPC clustering or its impact on the distribution or accessibility of chromatin in the yeast nucleus. Intriguingly, one recent report has shown that mutations in the chromatin remodeler RSC cause both severe defects in nuclear envelope morphology and mislocalization of nucleoporins to the nuclear interior, suggesting that defects in the structure and/or assembly of NPCs might be linked to changes in global chromatin state [49].

**Figure 8:**
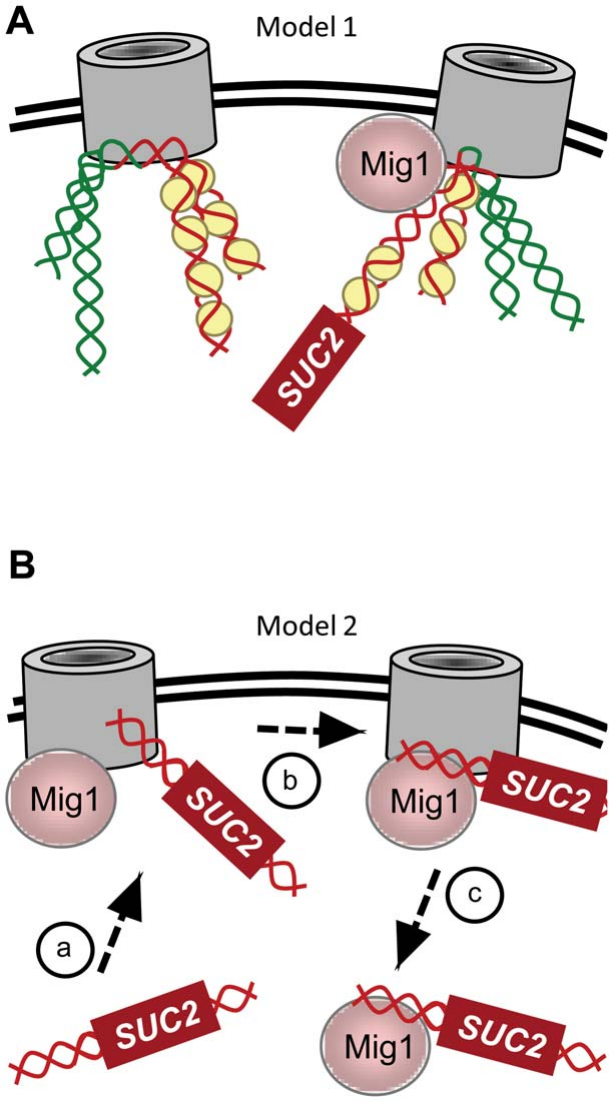
Two models for NPC-dependent Mig1 repression. (A) Model 1, NPCs mark transcriptional boundaries and help regulate nucleosome position. NPCs interact with chromatin, establishing boundaries between active (green) and inactive (red) portions of the genome (represented by four loci on two DNA molecules attached to each nuclear pore). These boundaries provide a register from which the fine-scale positioning of nucleosomes can be established (nuclear pore on the left). By accumulating in the perinuclear subcompartment during growth on glucose, Mig1 can easily find its site immediately after *SUC2* has visited the NPC and its promoter nucleosomes have been reset (nuclear pore on the right). In this model, deletion of either *NUP120* or *NUP133* disrupts nucleosome positioning throughout the genome, so that multiple Mig1 sites are masked and the repressor is blocked from binding DNA (not illustrated). (B) Model 2, NPCs facilitate DNA binding. (a) In the presence of glucose, Mig1 accumulates in the perinuclear subcompartment and *SUC2* makes transient contact with NPCs. (b) Increased local concentration of both the promoter and the repressor facilitates Mig1 binding to its consensus site upstream of *SUC2* and other target genes. (c) The repressed gene then moves back into the lumen, bound by Mig1. An alternative model not ruled out by the data presented here is that transient contact between Mig1 and the gene at NPCs is sufficient for repression. In this model, deletion of either *NUP120* or *NUP133* alters NPC structure in such a way that Mig1 can no longer associate, and thus can neither bind to DNA nor repress transcription from the promoters of glucose-repressed target genes. It should be noted that models (A) and (B) are not mutually exclusive.

We show here that removal of Nup120 or Nup133 results in the failure of Mig1 to occupy its consensus sites in target promoters (Fig. 6 and Fig. 7). This surprising finding explains the loss of *SUC2* repression in strains lacking either of these NPCs (Fig. 1B). Since Mig1 inhibits its own transcription [50], it may also explain the slight (approximately two-fold) increase in levels of the repressor protein in *nup120*Δ and *nup133*Δ mutants (Table 2). However, this discovery also raises an important new issue: why is Mig1 recognition of its specific binding sites in chromatin dependent on NPC subunits? The primary Mig1 site in the *SUC2* promoter is not normally covered by a nucleosome [51], [52], and there is no evidence that binding of the repressor is dependent on chromatin remodelers such as RSC. However, we cannot rule out the possibility that deletion of *NUP120* or *NUP133* alters chromatin structure in such a way as to stably reposition a nucleosome over the Mig1 site, thus blocking binding of the repressor to the DNA. Consistent with the suggestion of Titus *et al.* [49], this model implies that NPCs help to fine-tune nucleosome position throughout the genome, and in this way make a direct contribution to the regulation of transcriptional state (Fig. 8A). By associating with NPCs, then, Mig1 may be able to rapidly identify and associate with its target promoters immediately after nucleosomes have been precisely positioned to expose its binding site.

When Mig1 represses transcription, a substantial fraction of the protein co-purifies with NPCs (Figs. 3 & 4 and [21]); under these conditions, its canonical target gene *SUC2* can be seen periodically to visit the nuclear periphery, where its promoter physically interacts with NPCs [21]. Another possibility, then, is that Mig1 and the NPC bind DNA cooperatively (Fig. 8B). Indeed, if extended to other nuclear factors that recognize specific DNA motifs, this model represents a potential solution to an old conundrum concerning the kinetics of consensus site recognition in a typical eukaryotic genome; it is not clear how even DNA binding proteins with strong affinities for specific consensus sites (such as Gal4, with an equilibrium dissociation constant of 0.5 nM; [53], [54]) are capable of occupying target promoters in the eukaryotic nucleus due to the high concentration of non-specific DNA [55], [56], [57]. However, while facilitated DNA binding is common, so far neither the NPC nor any other structural feature within the nucleus have been found to mediate DNA binding.

Based on the data here, we cannot rule out the possibility that Nup120 and Nup133 are required for Mig1-mediated repression because these nucleoporins are important either for proper folding of the repressor or for post-translational modification of its DNA binding domain. An unequivocal test of this idea would be a direct assay of Mig1 DNA binding in *nup120*Δ and *nup133*Δ extracts; unfortunately *in vitro* DNA binding by Mig1 is detectable only at a high protein-to-DNA molar ratio, which requires overexpression of the repressor in *Escherichia coli* [9]. Nonetheless, we believe both these explanations to be unlikely. With respect to the former possibility, nucleoporins are not known to possess chaperone activity. Further arguing against this idea, misfolded proteins are usually targeted to proteasomes and degraded, whereas Mig1 levels in the *nup120*Δ and *nup133*Δ backgrounds are not reduced relative to the isogenic wild type. With respect to the latter possibility, there is also no evidence that nucleoporins mediate covalent modification of proteins. Moreover, post-translational modification of Mig1 appears to be limited to phosphorylation, which occurs only in the absence of glucose when the repressor is inactive and exported to the cytoplasm.

Although the work we present here represents an important advance in our understanding of how NPCs impact gene regulation, the multiple mechanisms depicted in Figure 7 highlight the need for a more precise definition of the roles these complex structures play in nuclear processes other than transport. In particular, this and other work suggests many interesting questions about the relationship between NPCs, chromatin architecture, nuclear organization, and transcription. For example, we have also recently found that NPCs interact with the canonical transcriptional activator Gal4 (our unpublished data); how common are such interactions, and how are they mediated? Is there a reciprocal relationship between NPC assembly and chromatin assembly? Since components of the NPC have been found to associate with numerous genomic loci in a variety of organisms [58], [59], [60], [61], [62], the answers to these questions are likely to reveal new and fundamental knowledge about gene regulation.

## Materials and Methods

### Strains, media, and assays

*S. cerevisiae* strains used in this study are listed in Table S1. All strains were grown in rich (yeast extract/peptone) media containing 2% glucose (repressing conditions) or 3% pyruvate (derepressing conditions). Invertase assays were done as described previously [63].

### Fluorescence microscopy

Localization of Mig1-GFP was visualized by using a Zeiss LSM 510 META confocal laser scanning microscope with a 63× Plan-Apochromat 1.4 NA Oil DIC objective lens. GFP was excited by using the 488 nm laser; emissions were detected with a 505–530 BP filter.

### Quantitative fluorescent protein detection (QFPD)

A Mig1-GFP strain from the Yeast GFP Clone collection (Invitrogen Life Technologies) was used as the starting material for QFPD experiments. PCR was used to confirm the correct integration of the GFP tag; PCR-mediated disruption was then used to generate isogenic mutant strains. Cytosolic, nuclear (nucleoplasmic/perinuclear) and perinuclear fractions were isolated from each strain and fluorescence was measured as previously described [21]. Briefly, nuclear and perinuclear fractions were isolated [64] and proteins of interest therein were detected [21], [30] as described previously. The yeast cell wall is digested to completion by incubation with a combination glusulase and zymolyase cocktail. The resulting spheroplasts are then resuspended in 1.1 M sorbitol, overlaid onto a Ficoll-sorbitol cushion, and centrifuged at 2000*g_max_* for 25 minutes; this step removes both the digestive enzymes and small buds, which do not lyse and would otherwise contaminate isolated nuclei. Purified spheroplasts in sorbitol are then immediately lysed in the presence of 5 mM DTT and protease inhibitor cocktail, using a Polytron homogenizer located in a 4°C cold room; the extent of lysis is monitored by examining 10 µL of this suspension under phase contrast microscopy. Spheroplasts are subjected to homogenization until less than 2% of cells remain unbroken and intact nuclei, which appear as small gray spheres, are visible. Lysed spheroplasts are then mixed with 0.6 M sucrose/polyvinylpyrollidone-40 (PVP-40) and centrifuged at 10,000*g* for 25 minutes at 4° to separate crude cytosol (supernatant) from intact nuclei (pellet). Once isolated, the pellet is resuspended in 2.1 M sucrose/PVP-40 and loaded onto a gradient consisting of 2.3 M, 2.1 M, and 2.01 M sucrose/PVP-40 steps. The loaded gradient is then centrifuged at 103,000*g* for 4 hours at 4°. After centrifugation, the first two layers of the gradient contain mitochondria, vesicles, and microsomes; the next two layers contain purified, intact yeast nuclei. To isolate NPCs, these intact nuclei are further centrifuged at 193,000*g* for 1 hour; after the spin, the supernatant is removed completely by aspiration. Buffer containing 0.01 M Bis-Tris pH 6.5, 100 mM MgCl_2_, 400 U/mL DNase I, 10 mM PMSF, and protease inhibitor cocktail is added to the nuclei, which are then immediately resuspended with vigorous vortexing sufficient to induce total lysis. Lysed nuclei are then incubated at room temperature for about ten minutes, until DNA is digested to completion. An equal volume of sucrose/Nycodenz solution (2.3 M sucrose, 0.24 M Nycodenz, 10 mM Bis-Tris pH 6.5, 100 nM MgCl_2_) is added to the lysed nuclei, and the mixture is overlaid first with 2.25 M sucrose/BT solution, then with 1.5 M sucrose/BT solution, and finally with BT solution (0.01 M Bis-Tris pH 6.5, 100 mM MgCl_2_) alone. The resulting gradients, containing the lysed nuclei in the bottom layer, are centrifuged at 103,000*g* for 24 hours at at 4°. NPCs, NPC-associated proteins, and nuclear membranes are found at the interface of the 1.5 M and 2.25 M fractions. This interface is recovered and then probed for proteins of interest, which were detected as previously described [21], [30]; Each fraction was aliquoted (0, 20, 40, 80 and 160 µg) into 96 well-plates for analysis with a Typhoon Phosphorimager (GE Healthcare). GFP was excited by using the 488 nm laser and the resulting fluorescence was acquired with the 526 short pass emission filter at high sensitivity with detection at +3 mm above the platen surface at 200 µm resolution. For quantitative analysis, densitometric values were obtained by using ImageQuant (GE Healthcare) and units of GFP per mg protein were determined. After detection, perinuclear: nucleoplasmic ratios were calculated and normalized to the corresponding values for the integral nuclear membrane protein Pom152, which was set to 100%.

### Co-immunoprecipitation and western blots

For immunoprecipitation, the starting Mig1-GFP strain described above was transformed with either Nup84-LexA, LexA alone, or empty vector. An anti-GFP antibody (Santa Cruz Biotechnology) was then used to pull down Mig1 according to a previously described protocol [39]. Both crude lysate and eluate were subjected to SDS-PAGE, followed by immunoblotting with an anti-LexA antibody (Santa Cruz Biotechnology). Immunodetection of HA-tagged Mig1 in different deletion backgrounds was done with an anti-HA (12CA5) antibody.

### Chromatin immunoprecipitation (ChIP)

ChIP assays were done as described previously [21]. Briefly, **c**hromatin extracts were prepared from TAP-tagged and HA-tagged strains (Open Biosystems) [52]. Immunoprecipitation was done with 5 µg of α-HA (12CA5 Roche) antibody and protein-A Sepharose beads. The final DNA pellet was resuspended in 30 µl TE; in all cases, 1, 2, and 3 µl were used for PCR amplification of target regions as a control for linearity of amplification. Products of approximately 250 bp were synthesized by using primers in the −200 to −850 bp region of each promoter. 20% of PCR products were resolved on 2% Nusieve agarose gels and imaged with a Chemidoc XRS (Biorad).

## Supporting Information

Figure S1 **Nup84-lexA fusions localize to the nuclear periphery.** Left panel shows confocal images of cells containing yellow fluorescent protein (YFP) fused to the C-terminus of lexA-tagged nucleoporin Nup84. The fusion proteins localize at the nuclear periphery, and are thus observed as distinct rings. Right panel shows the DIC images of the cells corresponding to the left panel.
(PDF)

Figure S2 **Exponential loss of** ***SUC2*** **repression upon depletion of Mig1 from the perinuclear compartment.** Perinuclear, lumenal, and total levels of nuclear Mig1-GFP were determined by QFPD analysis (y-axis); percent *SUC2* repression (x-axis) reflects the increase in invertase expression in nup mutants relative to wild type (see Fig. 4 and Table 2). The wild type data points are indicated by arrows.
(PDF)

Figure S3 **Binding of primers to the IP DNA is linear.** In wild type (WT) cells, Mig1 binds to the *SUC2* promoter in the presence of glucose (R; repressed conditions); in *snf1Δ* cells Mig1 binds to the *SUC2* promoter in both the presence and absence of glucose (D; derepressed conditions). Addition of increasing amounts of immunoprecipitated chromatin as template DNA (1X, 2X, 3X) produces a corresponding increase in the amount of PCR product.
(PDF)

Table S1 Yeast strains and plasmids used in this study. Strains are isogenic to BY263.
(XLSX)
