# Supplementary figures and images for: The Nuclear Pore Complex Mediates Binding of the Mig1 Repressor to Target Promoters
Source: PLoS One. 2011 Nov 14;6(11):e27117. doi: 10.1371/journal.pone.0027117 (PMC3215702; doi:10.1371/journal.pone.0027117)

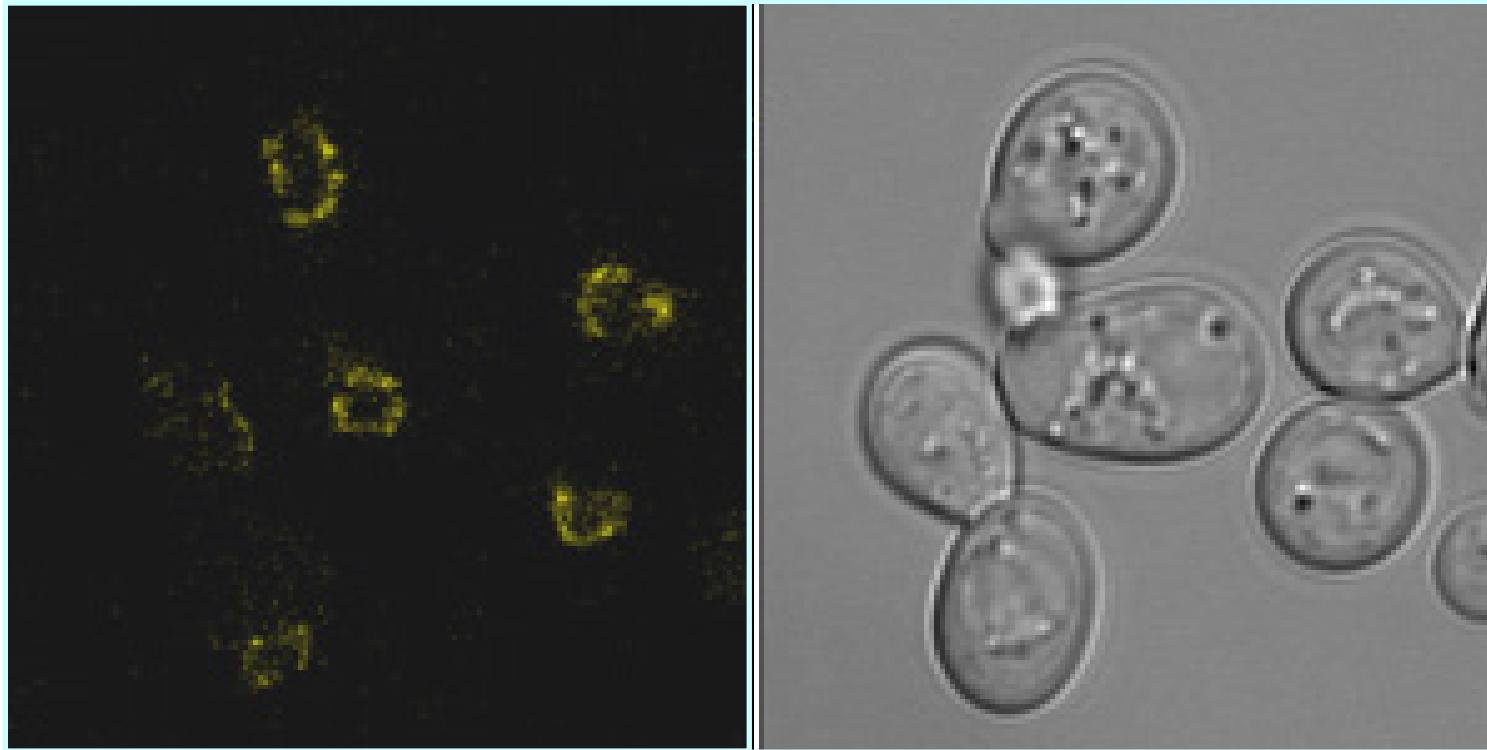

Supplemental Fig 1.

Supplement: Figure S1 — Nup84-lexA fusions localize to the nuclear periphery. Left panel shows confocal images of cells containing yellow fluorescent protein (YFP) fused to the C-terminus of lexA-tagged nucleoporin Nup84. The fusion proteins localize at the nuclear periphery, and are thus observed as distinct rings. Right panel shows the DIC images of the cells corresponding to the left panel. (PDF) [file pone.0027117.s001.pdf]

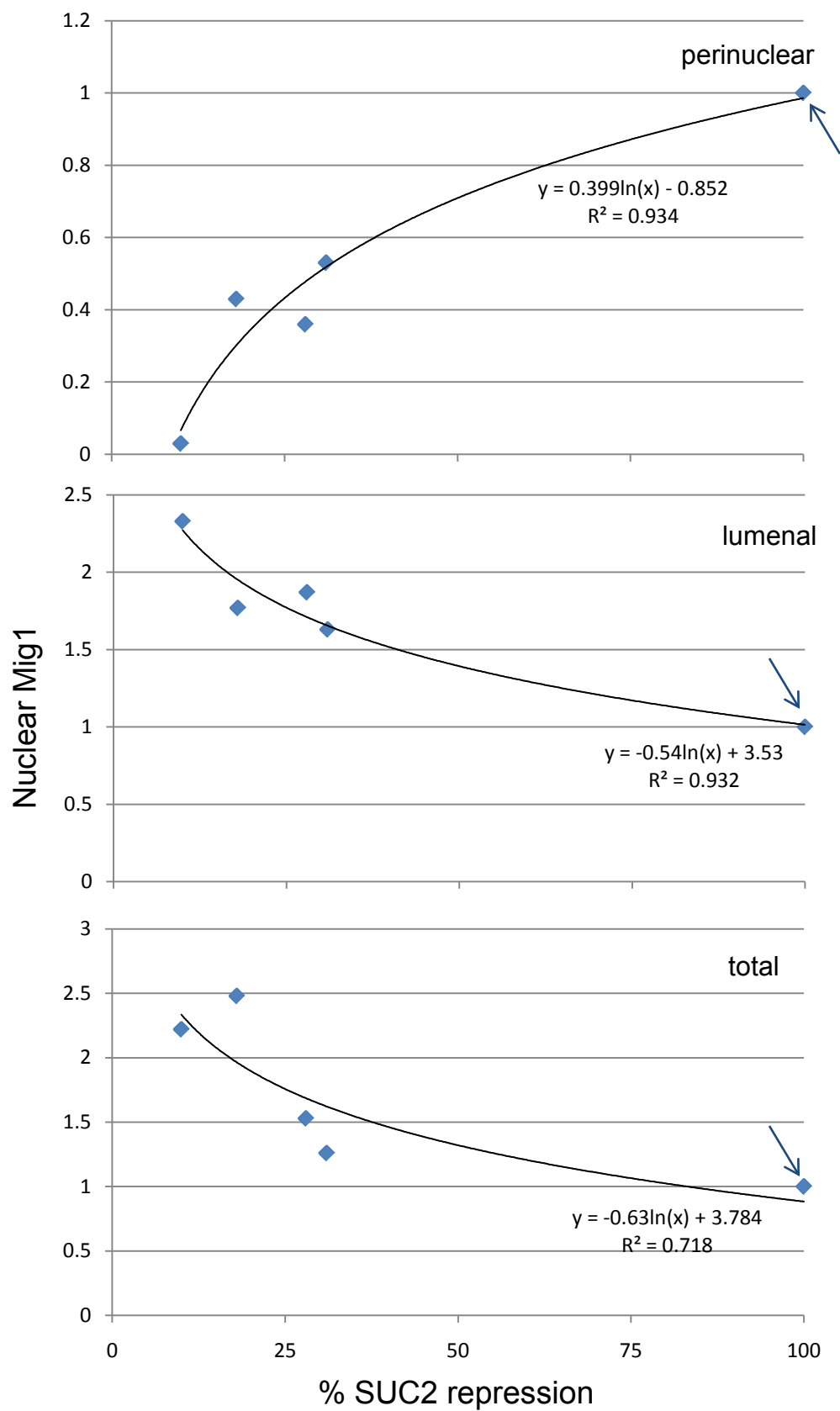

Supplemental Fig 2.

Supplement: Figure S2 — Exponential loss of SUC2 repression upon depletion of Mig1 from the perinuclear compartment. Perinuclear, lumenal, and total levels of nuclear Mig1-GFP were determined by QFPD analysis (y-axis); percent SUC2 repression (x-axis) reflects the increase in invertase expression in nup mutants relative to wild type (see Fig. 4 and Table 2). The wild type data points are indicated by arrows. (PDF) [file pone.0027117.s002.pdf]

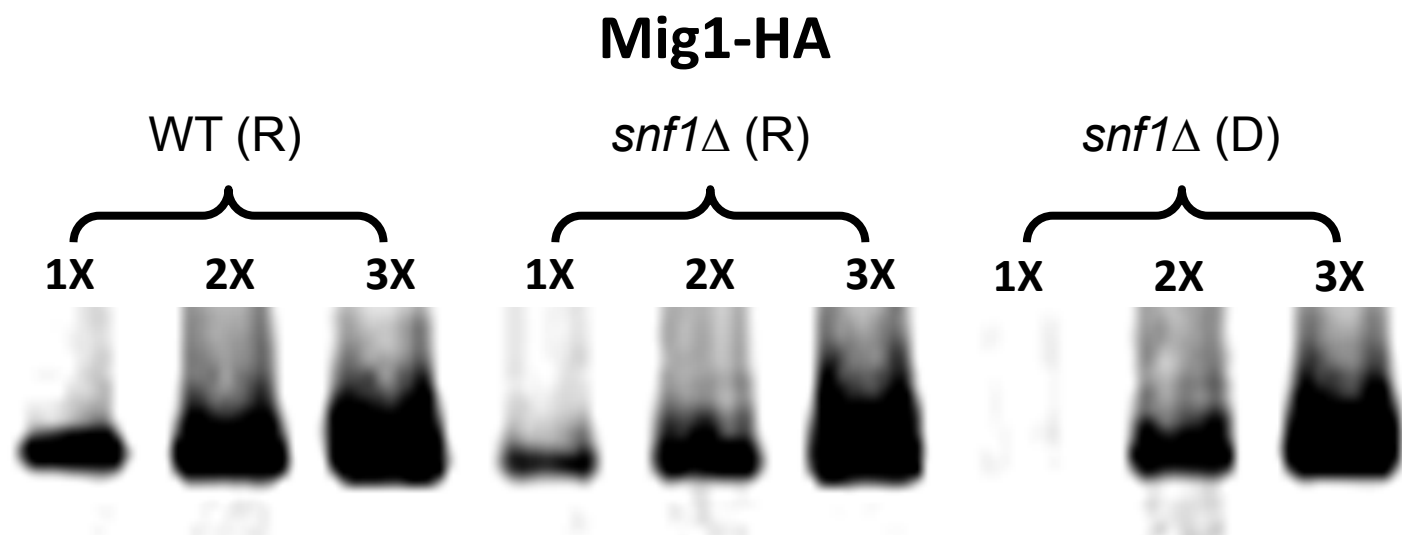

Supplemental Fig 3.

Supplement: Figure S3 — Binding of primers to the IP DNA is linear. In wild type (WT) cells, Mig1 binds to the SUC2 promoter in the presence of glucose (R; repressed conditions); in snf1Δ cells Mig1 binds to the SUC2 promoter in both the presence and absence of glucose (D; derepressed conditions). Addition of increasing amounts of immunoprecipitated chromatin as template DNA (1X, 2X, 3X) produces a corresponding increase in the amount of PCR product. (PDF) [file pone.0027117.s003.pdf]
